# Hot Gallbladders, Cool Outcomes: Bailout Outcomes of Early Laparoscopic Cholecystectomy for Acute Cholecystitis

**DOI:** 10.7759/cureus.88322

**Published:** 2025-07-19

**Authors:** Andrew C Ekwesianya, Farjana Enayathulla, Abraham Jesudoss, Abraham A Ayantunde

**Affiliations:** 1 Department of General and Colorectal Surgery, Southend University Hospital, Southend, GBR; 2 School of Medicine, Queen Mary University of London, London, GBR; 3 Department of General Surgery, Southend University Hospital, Southend, GBR

**Keywords:** acute calculus cholecystitis, bailout procedure, conventional laparoscopic cholecystectomy, gall bladder diseases and gallstones, gallbladder diseases and gallstones, interval laparoscopic cholecystectomy

## Abstract

Introduction

Laparoscopic cholecystectomy for acute cholecystitis (hot cholecystectomy) is known to be associated with increased operative difficulty and a higher risk of intraoperative biliovascular complications. Certain bailout strategies have been formulated to mitigate these risks. Most guidelines recommend surgery within seven days of onset of inflammation. The objective of this study is to assess the bailout rate of early cholecystectomy for acute cholecystitis, performed irrespective of the time of presentation but with a target of six weeks from the time of diagnosis.

Methods

This retrospective cohort study evaluated the outcomes of laparoscopic cholecystectomy over a three-year span (February 2022 to January 2025), comparing patients who underwent early laparoscopic cholecystectomy within six weeks of an acute cholecystitis diagnosis with those who underwent the procedure for a non-inflamed gallbladder. Data analysis was conducted using SPSS Statistics Version 29.0.1.0.

Results

A total of 355 patients (190 patients in the cholecystitis group and 165 patients in the non-cholecystitis group) underwent cholecystectomy during the period of review. The median duration of surgery was significantly longer in the treatment group than in the control group (80 vs 70 minutes; p = 0.007). There was no statistically significant difference in the rate of subtotal cholecystectomy (3.2% vs 0.6%; p = 0.605) or abandonment of surgery (2.6% vs 0.6%; p = 0.653) between the treatment and control groups. There were no conversions to open cholecystectomy, need for intraoperative percutaneous cholecystostomy or surgery-related mortality in either group. The overall bailout rate, however, was higher in the cholecystitis group (5.8% vs 1.2%, p = 0.039).

Conclusion

This study indicates that laparoscopic cholecystectomy is safe for acutely inflamed gallbladder even after the first week of onset of inflammation. While the overall surgical bailout rate was significantly higher in patients with acute cholecystitis, individual bailout outcomes were not significantly different from those in the non-cholecystitis group.

## Introduction

Gallstone disease is a common condition in surgical practice, affecting around 10% of the UK population in their lifetime [1]. Laparoscopic cholecystectomy is the gold standard treatment for symptomatic cholelithiasis, and around 0.12% of the English population undergo cholecystectomy annually [2].

Traditionally, cholecystectomy for acute cholecystitis was performed within the first few days of onset of inflammation; thereafter, the procedure would be deferred for 6 to 12 weeks to allow the inflammation to settle - a term known as delayed or ‘interval’ cholecystectomy. This approach was due to the recognition that acute cholecystitis increases the operative difficulty of the procedure and thus the risk of intraoperative biliovascular complications [3,4]. The UK National Institute for Health and Care Excellence (NICE) recommends that early laparoscopic cholecystectomy be performed within seven days of diagnosis of acute cholecystitis [5].

The Tokyo Guidelines classify acute cholecystitis into three grades - mild, moderate and severe - based on severity of the inflammation. According to the guidelines, duration of symptoms of cholecystitis greater than 72 hours is classed as moderate cholecystitis [6]. Earlier versions of the Tokyo Guidelines (TG07 and TG13) recommended early cholecystectomy only for mild cholecystitis (duration of symptoms less than 72 hours) [7,8]. However, the 2018 version of the Guidelines (TG18) recognises the safety of early cholecystectomy beyond 72 hours of onset of acute cholecystitis but recommends that it be performed in a tertiary hepatobiliary centre; otherwise, biliary drainage or delayed cholecystectomy should be performed [9].

In recognition of the risks associated with early cholecystectomy for acutely inflamed gallbladder (hot gallbladder), certain surgical bailout strategies have been advocated to mitigate the risk of biliovascular injuries during this procedure. These are alternative strategies that can be adopted in difficult gallbladder when complete cholecystectomy is not feasible without a high risk of biliary or vascular injury. The bailout techniques include abandonment of procedure, subtotal cholecystectomy, conversion to open cholecystectomy, intraoperative percutaneous cholecystostomy and retrograde cholecystectomy (fundus-first method) [10-12]. However, the fundus-first method has been described as an ‘error trap’ that may increase the risk of major biliary injuries during laparoscopic cholecystectomy, and thus is not a preferred bailout technique for many surgeons [13].

The aim of this study was to compare the outcomes of early cholecystectomy performed within six weeks of diagnosis of acute cholecystitis with the outcomes of cholecystectomy performed for non-inflamed gallbladder, evaluate the safety of early cholecystectomy and determine the rate of surgical bailouts of the procedure in a secondary, non-hepatobiliary centre in England.

## Materials and methods

Inclusion and exclusion criteria

The study included patients who underwent laparoscopic cholecystectomy for gallstone disease at Southend University Hospital, Essex County, England. The exclusion criteria were cholecystectomy for non-gallstone conditions, such as gallbladder dyskinesia, and patients with gallstone diseases that were deemed unfit for surgery due to frailty or the patient’s preference.

Diagnosis of acute cholecystitis was based on a combination of clinical findings (right hypochondrial tenderness), elevated inflammatory markers (white blood cell count and C-reactive protein) and radiological evidence of gallbladder inflammation (ultrasound scan, computed tomography [CT] scan or magnetic resonance cholangiopancreatography [MRCP]).

Study design

This is a retrospective cohort study that assessed the surgical bailout outcomes of patients who had early laparoscopic cholecystectomy within six weeks of diagnosis of acute cholecystitis at Southend University Hospital. Using the appropriate codes, the hospital’s database was searched to identify adult patients who were booked for ‘urgent’ or ‘very urgent’ laparoscopic cholecystectomy for acute cholecystitis over a three-year period (February 2022 to January 2025). These constituted the treatment (cholecystitis) group.

Data were also collected on patients who underwent laparoscopic cholecystectomy during the same timeframe for reasons other than cholecystitis, such as biliary colic or gallstone pancreatitis. These patients were designated as the control (non-cholecystitis) group. The urgency of the procedure was determined using a locally developed ‘traffic light’ scoring system.

The traffic light system is a preoperative assessment tool for the prediction of intraoperative surgical difficulties during laparoscopic cholecystectomy. It utilises patients’ demographics, body mass index, co-morbid medical conditions, derangements in liver function tests, imaging findings (ultrasound, CT, MRCP, endoscopic ultrasound, hepatobiliary iminodiacetic acid scan) and identified gallstone complications present in the patient. Weighted points are given to each of the parameters based on its adjudged contribution to intraoperative surgical difficulty. For example, uncomplicated acute cholecystitis has a score of 2, while the presence of pericholecystic abscess gives it a score of 4.

The total score obtained in an individual patient falls within either of three grades designated by the colours of the traffic light: colour green represents a total score of 0-3, amber a total score of 4-6 and red a total score of 7 or more, in increasing order of predicted surgical complexity. The difficulty grade also provides a useful guide to the theatre booking clerk in terms of the number of procedures booked for each theatre session, based on the anticipated surgical difficulty. Green patients are allotted an operating time of 90 minutes, amber an operating time of 120 minutes and red a surgical time of 180 minutes.

Additional information, including duration of surgery, use of drains and any bailout procedures undertaken, was sought for in the operation notes and anaesthetic charts. The operative difficulty was assessed and graded using the Nassar difficulty grading scale for cholecystectomy [14].

Data analysis

Data analysis was conducted using SPSS Statistics Version 29.0.1.0 (IBM Corp., Armonk, NY). Test of significance for categorical variables was conducted using Pearson’s chi-square tests at 95% confidence interval, with a p-value of <0.05 indicating a statistically significant difference between the variables. The independent t-test was used to compute the difference in mean scores between continuous variables.

## Results

A total of 355 patients were enrolled in this study. In the treatment group, 190 patients underwent early laparoscopic cholecystectomy within six weeks of diagnosis of acute cholecystitis. The age range was 22-96 years, with a median of 54 years. Sixty of the patients were males, giving a male-to-female ratio of 1:2.2. The duration of surgery ranged from 25 to 210 minutes, with a median of 80 minutes. Of the 190 patients, four (2.1%) underwent surgery performed independently by a trainee, while the remainder underwent surgery performed or assisted by dedicated consultant surgeons.

During the same period in review, 165 patients underwent laparoscopic cholecystectomy in the control group for non-inflammatory gallbladder conditions. There were 40 males, with a male-to-female ratio of 1:3.2. The median duration of cholecystectomy for non-inflamed gallbladder was significantly less than that for inflamed gallbladder (p = 0.007). The demographic characteristics of the treatment (cholecystitis) group and the control (non-cholecystitis) group, including the corresponding p-values, are outlined in Table 1.

**Table 1:** Study characteristics in both groups ^§^Independent t-test. ^◊^Pearson’s chi-square test. A p-value of <0.05 indicates a statistically significant difference between variables.

Variable	Cholecystitis Group	Non-Cholecystitis Group	p-Value
Total number of patients	190	165	-
Age range in years (median age)	22–96 (54)	20–83 (46.5)	0.014^§^
Male:female	1:2.2	1:3.1	0.162^◊^
Duration of surgery in minutes (median duration)	25–210 (80)	25–190 (70)	0.007^§^
Mean Nassar intraoperative difficulty score	2.35	1.68	<0.001^§^
Frequency of drain use (percent)	15 (7.9%)	5 (3.0%)	0.054^◊^
Mean hospital stay in days	0.48	0.46	0.830^§^
Day cases (percent)	145 (76.3%)	134 (81.2%)	0.313^◊^
30-day readmission (percent)	5 (2.6%)	6 (3.6%)	0.558^◊^

A total of 157 (82.6%) patients in the treatment group underwent surgery after the first presentation of cholecystitis, while 24 (12.6%) underwent surgery following the second inflammatory episode (Table 2).

**Table 2:** Number of hospital presentations prior to surgery

Number of Presentations	Frequency	Percent
1	157	82.6
2	24	12.6
3	6	3.2
4	1	0.5
5	1	0.5
7	1	0.5
Total	190	100.0

**Figure 1:**
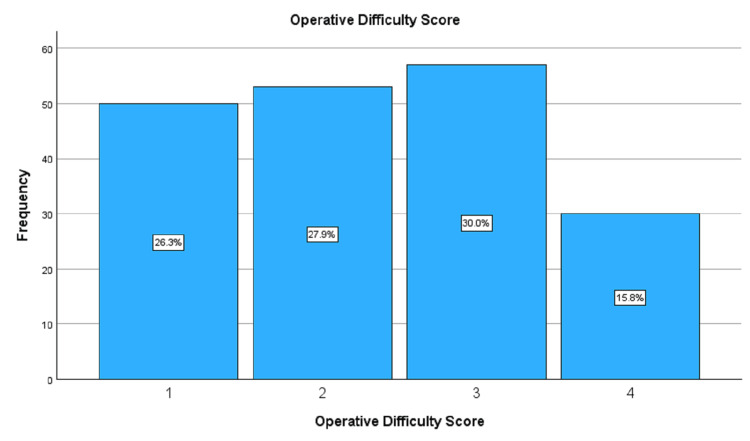
Bar chart showing distribution of the Nassar intraoperative difficulty scores of the patients with cholecystitis

On the Nassar operative difficulty scale for cholecystectomy, out of the 190 patients in the treatment group, 57 (30.0%) had grade 3 and 30 (15.8%) had grade 4 difficult cholecystectomy (Figure 1). The mean intraoperative difficulty score was 2.35. Intraoperative cholangiogram was performed in only two (1.1%) patients.

In the treatment group, 145 (76.3%) patients underwent operation as a day case, while 33 (17.4%) patients were discharged after an overnight stay (Table 3).

**Table 3:** Duration of postoperative hospital stay

Duration (Days)	Frequency	Percent
1	145	76.3
2	33	17.4
3	3	1.6
4	3	1.6
5	3	1.6
7	1	0.5
9	1	0.5
13	1	0.5
Total	190	100.0

Six (3.2%) patients in the treatment group required subtotal cholecystectomy, while five (2.6%) had the procedure abandoned due to severe inflammation and adhesions (Table 4).

**Table 4:** Reasons for abandonment of surgery GB, gallbladder

Patient	Study Group	Indication for Surgery	Reason for Abandonment
Patient 1	Treatment group	Acute calculous cholecystitis	GB empyema, obscured Calot’s triangle, extensive adhesions with duodenum and transverse colon
Patient 2	Treatment group	Acute calculous cholecystitis	Inflammatory phlegmon containing the omentum, transverse colon and GB
Patient 3	Treatment group	Acute calculous cholecystitis	Extensive adhesions involving the GB and small bowel loops
Patient 4	Treatment group	Acute-on-chronic cholecystitis	Chronic cholecystitis with complete tethering of duodenum to the GB
Patient 5	Treatment group	GB perforation with pericholecystic abscess.	Contracted GB, with the duodenum densely adherent
Patient 6	Control group	Gallstone pancreatitis; previous laparotomy for bowel obstruction	Extensive adhesions involving the GB, omentum and colon

There were no conversions to open cholecystectomy or need for intraoperative percutaneous cholecystostomy. The overall bailout rate, 11 (5.8%), was significantly higher in the patients who had cholecystitis than in the non-cholecystitis group (p = 0.039); however, the individual bailout outcomes were not statistically different between the two groups. As shown in Table 5, there was no statistically significant difference in the rate of subtotal cholecystectomy (p = 0.133) or abandonment of surgery (p = 0.147) between the treatment and control groups, with no surgery-related mortality.

**Table 5:** Comparative outcomes of bailout procedures between the two groups ◊Pearson’s chi-square test. A p-value of <0.05 indicates a statistically significant difference between variables.

Bailout Technique	Cholecystitis Group	Non-Cholecystitis Group	p-Value
Subtotal cholecystectomy	6 (3.2%)	1 (0.6%)	0.133^◊^
Abandonment of surgery	5 (2.6%)	1 (0.6%)	0.147^◊^
Conversion to open cholecystectomy	0	0	-
Cholecystostomy drain	0	0	-
Overall bailout rate	11 (5.8%)	2 (1.2%)	0.039^◊^
Mortality	0	0	-

In the cholecystitis group, three patients were readmitted for wound infection, one patient for bile leak (managed by radiologically-guided percutaneous drainage), and one patient due to significant postoperative pain. There was no postoperative complication that warranted a return to theatre in both groups.

## Discussion

The safety of early laparoscopic cholecystectomy for cholecystitis has been a subject of discussion for decades. Some studies have reported that delaying cholecystectomy beyond the first 24-72 hours of onset of cholecystitis is associated with a significantly increased incidence of bile leaks [15]. In the analysis of data from the Swedish Registry of 15,760 cholecystectomies performed for acute cholecystitis and 71,348 for non-inflamed gallbladder from 2006 to 2014, intraoperative and postoperative adverse events were significantly higher if the procedure was performed after four days of onset of acute cholecystitis; the optimum timing for cholecystectomy was reported to be within one to two days of presentation with cholecystitis [16]. The Tokyo Guidelines recommend that early cholecystectomy performed outside this ‘window’ period should be done in a specialist hepatobiliary centre [9]. Given the rising number of patients with symptomatic acute gallstone disease seen by general surgical teams, some surgeons in non-tertiary centers have developed the expertise to manage these cases, achieving outcomes comparable to tertiary hepatobiliary centers in carefully selected patients.

Application of this narrow ‘safety window’ for most patients with cholecystitis is difficult due to busy emergency theatre schedules, resource constraints and delayed presentation to hospital by many patients. The concept of interval cholecystectomy allows time for the inflammation to subside before cholecystectomy is performed to reduce the risk of these perioperative adverse events. The World Society of Emergency Surgery, in the 2020 updated guidelines for acute cholecystitis, recommended that delayed cholecystectomy be performed after six weeks from the first clinical presentation if early cholecystectomy cannot be performed within seven days of hospital admission or within 10 days of onset of symptoms. However, the authors recognised that this opinion was based on a very low quality of evidence and a weak strength of recommendation [17].

Interval cholecystectomy entailed that patients who present to the hospital after the early cholecystectomy window period would have to wait for weeks to months to undergo the procedure. This strategy is unfortunately associated with its own problems, including the risk of repeated attacks of biliary colic and inflammation during the waiting period; risk of choledocholithiasis, cholangitis and gallstone pancreatitis; increased waiting list for cholecystectomy; and the attendant cost of repeated hospital admissions [18,19].

In our hospital, patients who have a diagnosis of acute calculous cholecystitis and are fit for surgery are booked on the urgent or very urgent list for ‘hot’ laparoscopic cholecystectomy, and the procedure is performed by recognised and dedicated consultant surgeons irrespective of the time of presentation but with a target of six weeks from the time of diagnosis and/or presentation. This study, therefore, explored the outcome of this approach of early laparoscopic cholecystectomy on the surgical bailout rate in a non-hepatobiliary secondary care hospital.

Our cohort study compared the outcomes of early laparoscopic cholecystectomy for ‘hot gallbladder’ against routine and/or elective cholecystectomy for non-inflamed gallbladder to determine any significant difference in the surgical bailout rates. There was a significant difference (p = 0.007) in the duration of cholecystectomy between the cholecystitis group and the control group, reaffirming the fact that cholecystitis increases the difficulty of the surgery and thus prolongs its duration. This knowledge has helped us in time and resource allocation in our early cholecystectomy service delivery in the hospital.

There was a significant difference (p = 0.014) in the age of patients in the gallstone cholecystitis group (median age 54.0 years) compared to the non-cholecystitis group (median age 46.5 years). This suggests that the occurrence of cholecystitis happens several years after the initial formation of the gallstones. However, there was no significant difference in sex distribution (p = 0.162), duration of postoperative hospital stay (p = 0.830), use of wound drain (p = 0.054), day case rate (p = 0.313) and 30-day readmission rate (p = 0.558) between the two study groups, indicating a close similarity in their postoperative outcomes.

Nearly half (45.8%) of the patients in the treatment group had a Nassar cholecystectomy difficulty score of 3 or 4, reflecting the severity of the inflammation in this class of patients. These difficulties resulted in a significant increase in the overall bailout rate between the two groups (p = 0.039). However, a subgroup analysis of individual bailout outcomes did not show any significant difference in the rates of subtotal cholecystectomy (p = 0.133) and abandonment of surgery (p = 0.147) between the cholecystitis and non-cholecystitis groups.

The most common reasons for abandonment were severe inflammation obscuring the Calot’s triangle and dense adhesions involving the omentum, duodenum and transverse colon. There was no incidence of conversion to open procedure or intraoperative cholecystostomy drain insertion in both groups. We believe that we were able to achieve these outcomes because of the fact that early cholecystectomy is performed by experienced consultant surgeons and their trainees with particular interest and expertise in this area.

In a systematic review of discordant meta-analyses on early versus delayed cholecystectomy for acute cholecystitis, Song et al. also found no significant difference in the mortality, bile duct injury and conversion to open surgery between the two groups. The authors recommended early laparoscopic cholecystectomy to be the standard treatment option for acute cholecystitis [20].

Limitations

This study, like other retrospective cohort studies, was non-randomised and depended only on the parameters that were available in our hospital records. In our hospital, patients with severe forms of cholecystitis, such as perforated gallbladder, not improving on antibiotic treatment are generally managed by radiology-guided percutaneous cholecystostomy during the initial hospital admission, with the exception of a few who have emergency cholecystectomy at presentation if there is an availability of theatre space and a dedicated consultant surgeon. Some others who were deemed unfit for anaesthesia were not booked for surgery, with the implication that the surgical bailout rate might have been affected if they were all operated on routinely as other patients.

The few patients who had severe cholecystitis that posed significant risks of intraoperative biliovascular injuries and thus were abandoned instead of conversion to open cholecystectomy were eventually referred to dedicated upper gastrointestinal surgeons who were able to successfully perform some of the procedures laparoscopically without having to convert to open cholecystectomy. Nonetheless, this does not diminish the vital role of open cholecystectomy in managing complex cases, particularly when intraoperative complications arise.

## Conclusions

The traditional approach of interval (delayed) cholecystectomy for the management of acute cholecystitis is increasingly becoming obsolete. While acute cholecystitis is known to increase the intraoperative difficulty during cholecystectomy and thus the risk of perioperative complications, interval cholecystectomy increases the potential for recurrent presentations with acute gallstone complications. The results of this study indicate that performing early cholecystectomy in an acutely inflamed gallbladder increases the overall surgical bailout rate when compared to routine cholecystectomy for non-inflamed gallbladder, but this has no impact on the rate of same-day discharge and overall postoperative outcome, when performed by dedicated non-hepatobiliary surgeons.
